# Supplementary material for: Osteoprotegerin genetic polymorphisms and their influence on therapeutic response to ibandronate in postmenopausal osteoporotic females
Source: PLoS One. 2023 Sep 26;18(9):e0291959. doi: 10.1371/journal.pone.0291959 (PMC10521989; doi:10.1371/journal.pone.0291959)
Supplement: Attachment — Submitted filename: Plos1 .docx [file pone.0291959.s002.docx]

Osteoprotegerin genetic polymorphisms and their  influence on therapeutic response to ibandronate in postmenopausal osteoporotic females

The authors present interesting data re the association of different SNPs in osteoprotegerin (OPG) with the therapeutic response to ibandronate in postmenopausal osteoporotic.

1- Thorough language and grammatical proofing are required.
2- No sample size is done.
3- In Case-control study the number of controls should be greater or equal to the test subjects.
4- Preparation of samples prior to sequencing is missing (used primers are not written)
5- Haplotype analysis is recommended.
