# Supplementary material for: Osteoprotegerin genetic polymorphisms and their influence on therapeutic response to ibandronate in postmenopausal osteoporotic females
Source: PLoS One. 2023 Sep 26;18(9):e0291959. doi: 10.1371/journal.pone.0291959 (PMC10521989; doi:10.1371/journal.pone.0291959)
Supplement: Attachment — Submitted filename: Response to reviewers.docx [file pone.0291959.s003.docx]

Reviewer 1:

**Query:** The authors performed a case-control study in order to verify a possible association with OPG SNPs in menopause women before and after ibandronate therapy and compare with healthy women from Pakistan. Although the authors did not selected SNPs according population frequency or direct function upon OPG levels, the investigation has scientific and clinical importance. Still, they did not associated genotypes for analysis. Another important issue is the fact that there is no association with the SNPs per se but due population intrinsical variations. The conclusion is written in a confounding manner indicating a dubious association.

**Response:**

We sincerely appreciate the time and effort you dedicated to reviewing our manuscript. We acknowledge the concerns raised regarding certain aspects of our study, and we have carefully considered each of them.

We thank you for highlighting this point. While we agree that selecting SNPs based on population frequency or direct function upon OPG levels could have been beneficial. Gene was selected on the basis of the most reported genetics association with osteoporosis from National Center for Biotechnology Information (NCBI) (http://www.ncbi.nlm.nih.gov). Those SNPs were selected which were involved either directly or indirectly in disease pathogenesis, bone degradation and inflammation. Our intention was to conduct an exploratory investigation to identify potential associations that could be further validated as these SNP’s were the first to study in Pakistani population.

We acknowledge that there may be population-specific intrinsic variations contributing to the associations observed. Our study was conducted with menopausal women from Pakistan, and we agree that including additional population cohorts for validation would strengthen the robustness of our findings. In future research, we will make efforts to collaborate with researchers in other regions to encompass a more diverse population in our study.

Conclusion has been rewritten to remove ambiguity and is highlighted in yellow within the manuscript.

Reviewer #2:

**Query:** In the current study, Tariq et al. analyzed the association of the influence of OPG polymorphisms on ibandronate therapy. This study has some clinical implications, but it looks like the study is incomplete. Studying OPG without RANK and RANKL cannot provide a trustable conclusion. Authors should also add the most common SNPs of RANK and RANKL genes and establish a correlation with therapeutic response.

It needs to be clarified why the authors have chosen three SNPs and ignored several other very important and common SNPs; better add more SNPs.

**Response:**

In our population, there is a lack of information evaluating the relationship between polymorphisms of the OPG gene and bone mineral density. Moreover, there is no published data available describing the single nucleotide polymorphisms (SNP) of the OPG gene and its association with osteoporosis. Those SNPs were selected which were involved either directly or indirectly in disease pathogenesis, bone degradation and inflammation. Our intention was to conduct an exploratory investigation to identify potential associations that could be further validated as these SNP’s were the first to study in Pakistani population. Increase in SNPs also increases the financial burden. We are thankful to the reviewer for highlighting the importance of studying RANK and RANKL genes along with OPG. We acknowledge that the interaction of OPG, RANK, and RANKL is crucial in bone metabolism. In this study, we focused on OPG polymorphisms, therefore we didn’t explore RANKL.

**Query:** It would have been better if the authors performed gene seq and identified novel mutations or SNPs prevailing in the Pakistani therapeutic response. Pakistan has a high ratio of consanguineous marriages, thus higher chances of hereditary osteoporosis; did authors consider analyzing the inheritance pattern of osteoporotic genes?

**Response:**

We greatly appreciate your suggestion and recognize the significance of gene sequencing in future studies focusing on osteoporosis and therapeutic response in populations with a high prevalence of consanguineous marriages. This has been incorporated in limitation section and highlighted in yellow.

**Query:** Why only ibandronate?

**Response:**

Ibandronate is a long-acting bisphosphonate which is usually given once a month and is indicated for treatment as well as prevention of postmenopausal osteoporosis. Ibandronate is preferred as it is given once a month, and patient compliance is better, which means that it is easy for him to stick to the once-a-month regimen as compared to once-weekly alendronate. Adherence to the treatment is important to improve patient outcomes. It also decreases the economic and social burden of the disease

Inderjeeth, C.A., Glendenning, P., Ratnagobal, S.,Inderjeeth, D.C. and Ondhia, C., 2015. Long-term efficacy, safety, and patient acceptability of ibandronate in the treatment of postmenopausal osteoporosis. Int. J. Women's health, 7:7.

**Query:** The entire manuscript requires thorough revision for language improvement. There are a lot of grammatical and other language errors.

**Response:**

We have taken the help of professional editing services for language editing of the manuscript.

**Query:** Please avoid self-citations; the authors have cited a bunch of their other articles.

**Response:**

You are right that we should avoid self-citations, but we have been working on osteoporosis for the last decade and have published many osteoporosis-related studies. Therefore, we cited our few related articles. However, we have removed two references (36 and 38) on your recommendation.

**Reviewer 3**

**Query:** In introduction:
-Please write the full terminology of OPG in its first appearance in text as well as BMD and BMI.

**Response:**

Done and highlighted in yellow
**Query:** -In line 73-75, please remove G1181C as you are mentioning that G allele increases osteoporosis and C allele in G1181C might decrease the risk of osteoporosis (in line 76-78).

**Response:**

Thank you for mentioning this. Done and highlighted

**Query:** In methods:

-Please mention the age of the control group

**Response:**

Done and highlighted

**Query:** -How was sample size calculated/determined?

**Response:**

The details have been mentioned in the methods.
**Query:** -Please clarify the sample size of the non-control and control group in Methods. Please write the correct sample size of the control as it was written 40 in methods and 46 in results section.

**Response:**

Sample size calculation and correction has been added in the manuscript and highlighted in yellow.

**Query:** -Please write the manufacturer details of the kits used to measure OPG and the kits used to extract DNA.

**Response:**

Added and highlighted in the manuscript

**Query:** -Please give more details about DEXA including the machine used and the bone sites evaluated as well as the criteria used to evaluate BMD.

**Response:**

Added and highlighted

**Query:** -Please give more details about SNPs sequencing including primer designs for each SNP.

**Response:**

Added and highlighted

**Query:** -Latest references related to the study need to be added in the revised manuscript.

**Response:** Five recent references have been added.

**Query:** There are some grammatical mistakes and need to be corrected by native speaker to improve the language and quality of the manuscript.

**Response:**

We have taken the help of professional editing services for language editing of the manuscript.

**Query:** The authors have to do BMD pre and post treatment which is necessity for this study. Moreover, the samples must be separated based on mild, moderate and severity too.
Hence, this study needs to be focused more on the research to get the outcomes.

**Response**

You are right that BMD should have been measured twice in patients (pre and post-treatment). We appreciate your suggestions. The literature indicates that just after six months, no significant change in BMD is expected (Please check following references). Additionally, DEXA is expensive in our country, so we avoided measuring DEXA. However, we have included this point in the limitation section (highlighted in yellow). We didn’t separate them because the sample size was not large enough to study them into subgroups (mild, moderate, and severe).

Rühling S, Schwarting J, Froelich MF, Löffler MT, Bodden J, Petzsche MR, Baum T, Wostrack M, Aftahy AK, Seifert-Klauss V, Sollmann N. Cost-effectiveness of opportunistic QCT-based osteoporosis screening for the prediction of incident vertebral fractures. Frontiers in Endocrinology. 2023;14:1222041.

Miller P D, McClung M R, Macovei L, et al. Monthly oral ibandronate therapy in postmenopausal osteoporosis: 1‐year results from the MOBILE Study. J Bone Miner Res. 2005;20:1315–1322.
