# Supplementary material for: Osteoprotegerin genetic polymorphisms and their influence on therapeutic response to ibandronate in postmenopausal osteoporotic females
Source: PLoS One. 2023 Sep 26;18(9):e0291959. doi: 10.1371/journal.pone.0291959 (PMC10521989; doi:10.1371/journal.pone.0291959)
Supplement: Attachment — Submitted filename: Response to reviwer comments R2.docx [file pone.0291959.s004.docx]

**Reviewer comments**

**Editor’s queries**

**Query 1**

Insert footnotes regarding the used test under tables 1 &2

**Response**

Done as suggested

**Query 2**

Write the strengths of the study

**Response**

Done as suggested

**Query 3**

Write the recommendation in the abstract section

**Response**

Done as suggested

**Reviewer** **queries**

The authors present interesting data on the association of different SNPs in osteoprotegerin (OPG) with the therapeutic response to ibandronate in postmenopausal osteoporotic.

**Response**

Thank you very much for liking our research.

**Query 2**

Thorough language and grammatical proofing are required.

**Response**

We got language editing from a professional editing agency.

**Query 3**

No sample size is done.

**Response**

The sample size calculation has been mentioned in detail in the methodology section.

**Query 4**

In a case-control study the number of controls should be greater or equal to the test subjects.

**Response**

Yes, you are right, but we could not keep them equal because of financial restraints. We have included this point in the limitation section.

**Query 5**

Preparation of samples prior to sequencing is missing (used primers are not written).

**Response**

The samples were sent to Advance Bioscience International (ABI) China for sequencing. The data obtained after sequencing was then visualized using Bio Edit Software and then analyzed using BLAST (NCBI) to find out the variations in these sequences. We have added this to the methodology section.

Already reported primers were chosen and rechecked with sequences from the NCBI website, while the longer sequence was obtained from the UCSC website (http://genome.ucsc.edu/). Primers' specificity was validated using the NCBI database of the human genome with BLAST (<http://www.ncbi.nlm.nih.gov/BLAST/>). This paragraph has been included in the methodology section. We selected three OPG gene SNPs: A163G, T245G, and G1181C.

**Query 6**

Haplotype analysis is recommended.

**Response**

Using the available data, our study design was structured to address specific research questions. Given the size of our study sample and the nature of our variables of interest, we determined that other statistical methods were more appropriate for addressing our research objectives. Haplotype analysis often requires substantial additional data, computational resources, and statistical expertise. Unfortunately, we could not undertake haplotype analysis within the scope of this study due to resource limitations, both in terms of data availability and analytical capacity.
